# Population of Naples

**Published:** 1831

**Authors:** 


Population of Naples.

The Osservatore Medico, for May, 1831, gives us rather a sorry account of the salubrity of Italy's boasted climate. It appears that the number of births in Naples, during the year 1830, was 14,267, while the deaths amounted to 15,419, leaving a deficit of 1152 in the population of the "finest climate in the world," in one year—and that year undistinguished by plague, pestilence, famine, or war! Our readers are aware that, according to Dr. Hawkins, the ratio of mortality in London is about 1 in 40 annually. In Naples during last year it was 1 in 23 and a fraction! What will the advocates of an Italian climate say to this? We believe, indeed, that the mania for running across the Alps and Apennines to bask in the brilliant suns of Italy, for restoration of health, is nearly over. It is the duty of medical men to make the non-professional public acquainted with the nature and effects of a transalpine climate, before their patients incur the fearful risk and expense of foreign travel — or rather of foreign residence. The British Isles offer facilities for travelling exercise in the Autumn far superior to the Continent — and perhaps equally conducive to health, at a much less expenditure of time and money. The day is probably not far distant when Snowdon and Ben Nevis will attract more tourists than the Simplon and the St. Bernard — when Windermere, Killarney, and Loch Lomond will vie with Como, Lake Leman, and the Lago Maggiore. In the former routes, as much health may probably be gained, and far less money and morality expended in the pursuit!

